# Antibiotic Therapy in the Treatment of COVID-19 Pneumonia: Who and When?

**DOI:** 10.3390/antibiotics11020184

**Published:** 2022-01-31

**Authors:** Tat Ming Ng, Sean W. X. Ong, Audrey Y. X. Loo, Sock Hoon Tan, Hui Lin Tay, Min Yi Yap, David C. Lye, Tau Hong Lee, Barnaby E. Young

**Affiliations:** 1 Department of Pharmacy, Tan Tock Seng Hospital, 11 Jalan Tan Tock Seng, Singapore 308433, Singapore; tat_ming_ng@ttsh.com.sg (T.M.N.); audrey_YX_loo@ttsh.com.sg (A.Y.X.L.); sock_hoon_tan@ttsh.com.sg (S.H.T.); hui_lin_tay@ttsh.com.sg (H.L.T.); min_yi_yap@ttsh.com.sg (M.Y.Y.); 2 Department of Infectious Diseases, National Centre for Infectious Diseases, 16 Jln Tan Tock Seng, Singapore 308442, Singapore; sean_ongwx@ttsh.com.sg (S.W.X.O.); david_lye@ncid.sg (D.C.L.); tau_hong_lee@ncid.sg (T.H.L.); 3 Department of Infectious Diseases, Tan Tock Seng Hospital, Singapore 308433, Singapore; 4 Lee Kong Chian School of Medicine, Nanyang Technological University, Singapore 308232, Singapore; 5 Yong Loo Lin School of Medicine, National University of Singapore, Singapore 117597, Singapore

**Keywords:** antibiotics, COVID-19, pneumonia, bacterial infection

## Abstract

Background: COVID-19 imposes challenges in antibiotic decision-making due to similarities between bacterial pneumonia and moderate to severe COVID-19. We evaluated the effects of antibiotic therapy on the clinical outcomes of COVID-19 pneumonia patients and diagnostic accuracy of key inflammatory markers to inform antibiotic decision-making. Methods: An observational cohort study was conducted in patients hospitalised with COVID-19 at the National Centre for Infectious Diseases and Tan Tock Seng Hospital, Singapore, from January to April 2020. Patients were defined as receiving empiric antibiotic treatment for COVID-19 if started within 3 days of diagnosis. Results: Of 717 patients included, 86 (12.0%) were treated with antibiotics and 26 (3.6%) had documented bacterial infections. Among 278 patients with COVID-19 pneumonia, those treated with antibiotics had more diarrhoea (26, 34.7% vs. 24, 11.8%, *p* < 0.01), while subsequent admissions to the intensive care unit were not lower (6, 8.0% vs. 10, 4.9% *p* = 0.384). Antibiotic treatment was not independently associated with lower 30-day (adjusted odds ratio, aOR 19.528, 95% confidence interval, CI 1.039–367.021) or in-hospital mortality (aOR 3.870, 95% CI 0.433–34.625) rates after adjusting for age, co-morbidities and severity of COVID-19 illness. Compared to white cell count and procalcitonin level, the C-reactive protein level had the best diagnostic accuracy for documented bacterial infections (area under the curve, AUC of 0.822). However, the sensitivity and specificity were less than 90%. Conclusion: Empiric antibiotic use in those presenting with COVID-19 pneumonia did not prevent deterioration or mortality. More studies are needed to evaluate strategies to diagnose bacterial co-infections in these patients.

## 1. Introduction

The typical symptoms of coronavirus disease 2019 (COVID-19) pneumonia are fever, cough and dyspnea [1]. These symptoms may trigger clinicians to start empiric antibiotic treatment while waiting for diagnostic testing such as a SARS-CoV-2 polymerase chain reaction test, radiology and blood investigations. Even if COVID-19 is confirmed, empiric antibiotics may be continued pending further evaluation if the treating physician is not able to conclude that bacterial co-infections have been adequately excluded. However, this is uncommon, particularly in early COVID-19 infection. It was reported that the bacterial co-infection rate was 3.5% but that 71.9% of COVID-19 patients received antibiotics in a large meta-analysis of studies representing more than 3000 patients [2]. In a separate cohort study of 989 COVID-19 patients, 31 (3.1%) had community-acquired bacterial co-infections. Microbiology-proven infections only occurred in 74 of them. However, more than 80% of the cohort were treated with antibiotics [3].

As the COVID-19 pandemic continues and intersects with the seasonal epidemics of other respiratory viruses, widespread unnecessary antibiotic use will subject patients to the risks of adverse effects and propagation of antimicrobial resistance globally. It is difficult to differentiate between progressing COVID-19 illness and bacterial co-infection or superinfection and the practice of empiric antibiotic treatment might be influenced by experiences from influenza, where bacterial co-infection rates are reported to be 11–35% [4]. Current guidelines recommend empiric antibiotics only if bacterial infections are suspected in moderate COVID-19, while empiric antibiotics are routinely recommended for severe COVID-19 [5,6]. 

A more recently published guideline on antibacterial therapy in COVID-19 infections concluded that there were no studies that evaluated the effectiveness and safety of antibiotics in patients with proven or suspected bacterial pneumonia [7]. Research in this area can help influence appropriate antibiotic decision-making. In this study, we aimed to evaluate the frequency of bacterial co-infection among hospitalised patients with COVID-19 and the prevalence of antibiotic use in these patients. We will also evaluate the effects of antibiotics on clinical outcomes and identify laboratory investigations that could help in identifying bacterial co-infection in COVID-19. 

## 2. Methods

### 2.1. Patient Recruitment

We conducted a retrospective cohort study of all patients with confirmed COVID-19 infection, as defined by positive severe acute respiratory syndrome coronavirus 2 (SARS-CoV-2)-specific real-time reverse-transcriptase polymerase chain reaction (RT-PCR) assay, who were admitted to the National Centre of Infectious Diseases and Tan Tock Seng Hospital, Singapore, between 22 Jan 2020 and 15 Apr 2020. This study was approved by the Domain Specific Review Board (Ref: DSRB 2020/01122). In Singapore over the period of this study, all individuals with COVID-19, regardless of the severity of infection, were isolated in hospitals.

### 2.2. Data Collection

Clinical and laboratory data were collected from the electronic medical record using an anonymised standardised case report form adapted from the World Health Organization (WHO) and International Severe Acute Respiratory and Emerging Infection Consortium (ISARIC) case record form for emerging severe acute respiratory infections. Data were collected until discharge or death. Patients were grouped into mild, moderate, severe or critical severity depending on their worst clinical status throughout the entire admission. Severity stratification was based on the classifications as outlined in WHO clinical management guidelines, with mild being defined as no pneumonia or hypoxia; moderate defined as pneumonia with no hypoxia (oxygen saturation ≥95%); severe defined as pneumonia with respiratory distress or hypoxia; and critical defined as having acute respiratory distress syndrome (ARDS), sepsis, or septic shock [5]. All COVID-19 therapeutics date were also collected. 

### 2.3. Study Endpoints

To evaluate the prevalence of bacterial co-infection in these patients, all positive microbiology results and concomitant suspected or confirmed bacterial co-infections as documented by the treating doctor within 5 days of COVID-19 diagnosis in the clinical notes were recorded. To evaluate the prevalence of antibiotic use, patients were defined as receiving empiric antibiotic treatment for COVID-19 if antibiotics were started within 3 days of COVID-19 diagnosis and within 3 days of the first chest radiograph of pneumonia in those with COVID-19 pneumonia. Subsequently, all cases where antibiotics were continued without interruption for more than 2 days were recorded and cumulative days of therapy were counted

To address the impact of antibiotics where bacterial pneumonia is suspected in moderate and severe COVID-19, outcomes data were collected. [5,6,7] These were subsequent ICU admission more than 2 days after diagnosis of COVID-19 pneumonia, 30-day mortality and in-hospital mortality. Length of stay data were also included after excluding in-hospital deaths. Diarrhoea (≥3 stools a day) was attributed to antibiotic therapy if it was within 14 days from the start of antibiotics. 

### 2.4. Statistical Analysis

No sample size calculation was performed. For comparisons between groups receiving antibiotics versus no antibiotics, the Mann–Whitney U test was conducted for continuous variables and Chi-square test or Fisher’s exact test was used as appropriate for categorical variables.

A multivariate logistic regression analysis was performed to evaluate the effects of antibiotics on 30-day mortality and in-hospital mortality rates. Variables with *p* < 0.05 on univariate analysis were included in the model. Among the SARS-CoV-2 therapeutics, only remdesivir was included in the model [8]. At the time of the study, dexamethasone was not part of the local treatment protocols and none of the patients received tocilizumab. 

The diagnostic accuracy of white cell counts and C-reactive protein and procalcitonin levels was determined using the area under the curve (AUC) calculated by receiver operator characteristic analysis, while cut-offs for maximal sensitivity and specificity considerations were determined using the Youden Index [9]. All tests were two-tailed and *p*-values of <0.05 were considered statistically significant. Statistics were performed using STATA SE 15. We referred to the STROBE checklist for reporting of cohort studies (Supplementary Materials).

## 3. Results

### 3.1. Clinical Features

**Figure 1:**
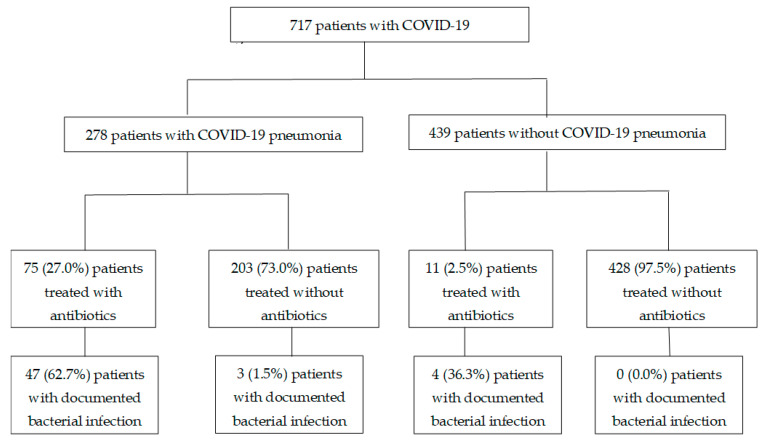
Flow diagram of COVD-19 patients who were treated with or without antibiotics. Documented bacterial infection: suspected or confirmed co-infections as documented within 5 days of COVID-19 (pneumonia) diagnosis.

Clinical data from 717 patients were collected. The median age was 46 (interquartile range (IQR) 29–57), 307 patients (42.8%) were female, and 401 (55.9%) were of Chinese ethnicity. In total, 156 patients (21.7%) had at least one co-morbidity and the median Charlson co-morbidity index value was 0 (IQR 0–0). The most common co-morbidities were diabetes mellitus (83, 11.6%), cardiovascular conditions (33, 4.6%), respiratory conditions (24, 3.4%) and malignancy (15, 2.1%). Regarding illness severity, 436 (60.8%) patients had mild illness throughout admission, 216 (30.1%) had moderate illness and 65 (9.1%) had severe or critical illness. There was a higher proportion of documented bacterial infections among patients in the ICU compared to non-ICU wards (10/30, 33.3% vs. 16/687, 2.3%). Similarly, more patients in the ICU were treated with antibiotics compared to non-ICU wards (24/30, 80.0% vs. 62/687, 9.0%). Overall, 278 (38.8%) of the COVID-19 patients had pneumonia, 86 (12.0%) were treated with antibiotics and only 54 (7.5%) had documented suspected or confirmed bacterial infections. Among those without pneumonia, only 11 (2.5%) were treated with antibiotics (Figure 1). 

### 3.2. Evaluation of Predictors for Bacterial Co-Infection

Compared to white cell counts and procalcitonin level, the C-reactive protein level had the best diagnostic accuracy for documented infections, with an AUC of 0.822 and 95%CI 0.756–0.887 in the overall cohort of patients and an AUC of 0.720 and 95%CI 0.633–0.808 for patients diagnosed with COVID-19 pneumonia. However, the sensitivity and specificity for C-reactive protein were below 90% for both groups of patients. The diagnostic accuracy of procalcitonin levels at a cut-off of >0.11 ng/mL was poor with AUC of less than 0.700, while sensitivity and specificity were less than 60% and 83%, respectively (Table 1).

### 3.3. Antibiotic Use

Among patients with COVID-19 pneumonia, 75 (27.0%) were treated with antibiotics. Fifty (18.0%) patients with COVID-19 pneumonia were documented to have concomitant bacterial infections. The proportion of these infections was statistically higher in those with higher CURB-65 scores (CURB-65 ≥ 3; 53.3%, 8/15 vs. CURB-65 0–2; 6.6%, 46/702). The documented sources of bacterial infections were respiratory tract (30/50, 60.0%), unknown sepsis (13/50, 26.0%), urinary tract, (3/50 6.0%), intra-abdominal (2/50, 4.0%), skin and soft tissue (2/50, 4.0%) and intravascular-catheter-related (1/50, 2.0%). Only 18 (6.6%) patients had positive microbiology. Respiratory cultures in 6 patients isolated *Candida tropicalis n = 1, C. albicans n = 2, Enterobacter aerogenes n = 1, Enterobacter cloacae n = 1 and Klebsiella pneumoniae n = 1*. Blood cultures in 5 patients isolated *Staphylococcus lugdunensis n = 1, coagulase negative Staphylococcus n = 2, Escherichia coli n = 1 and Enterobacter cloacae n = 1*. Urine cultures in 6 patients isolated *Proteus mirabilis n = 2, Klebsiella pneumoniae n = 1, Escherichia coli n = 1* and mixed growth *n = 2*. One patient had skin flora isolated from skin bullous fluid and 1 patient had a stool culture that isolated *Strongyloides stercoralis*. The median duration of antibiotic therapy was 8 (interquartile range, IQR 6–13) days. The majority of these patients were treated with amoxicillin-clavulanate (57/75, 76.0%), piperacillin–tazobactam (21/75, 28.0%), macrolides (35/75, 46.7%), carbapenems (14/75, 18.7%), cephalosporins (11/75, 14.7%) and fluoroquinolones (6/75, 8.0%) during their course of COVID-19 pneumonia. 

Patients treated with antibiotics were older with a higher median age (60 (inter-quartile range, IQR 51–69 vs. 55 (IQR 43–64), *p* = 0.008). They were also more likely to have higher peak C-reactive protein levels (122.5 (7.9–240.5) vs. 37.5 (IQR 8.4–77.7) mg/L, *p* = 0.008), be admitted to the ICU at the time of COVID-19 pneumonia diagnosis (26, 34.7% vs. 6, 3.0%, *p* < 0.001) and have received mechanical ventilation (13, 17.3% vs. 1, 0.5% *p* < 0.001). More patients who needed supplementary oxygen were treated with antibiotics (36, 48.0% vs. 26, 12.8%, *p* < 0.001). Although the median fraction of inspired oxygen was 21% (i.e., on room air) and similar between those treated without and with antibiotics, the upper quartile of those not treated with antibiotics was 21% compared to 36% in those treated with antibiotics. Patients treated with antibiotics were more likely to have documented bacterial infections during COVID-19 pneumonia treatment (4, 62.7% vs. 3, 1.5%, *p* < 0.001) and diarrhoea within 14 days of antibiotic treatment (26, 34.7% vs. 24, 11.8%, *p* < 0.001) (Supplementary Table S1).

Patients who were treated with antibiotics did not have a lower rate of subsequent admission to the ICU after diagnosis of COVID-19 pneumonia (6, 8.0% vs. 10, 4.9%, *p* = 0.384). However, 30-day mortality (10, 13.3% vs. 1, 0.5%, *p* < 0.001) and in-hospital mortality (10, 13.3% vs. 3, 1.5%, *p* < 0.001) rates were higher in those treated with antibiotics. Among patients who survived at hospital discharge, the median length of stay of those treated with antibiotics was longer (15, IQR (10–25) vs. 12, IQR (7–16), *p* < 0.0012) (Supplementary Table S1).

Multivariate analysis showed that antibiotic therapy was not independently associated with lower 30-day (adjusted odds ratio, aOR 14.492, 95% confidence interval, CI 0.533–393.875) or in-hospital mortality (aOR 3.690, 95% CI 0.240–56.811). Age and C-reactive protein levels were independently associated with higher 30-day and in-hospital mortality rates (Table 2 and Table 3).

## 4. Discussion

Among patients who received antibiotics for COVID-19 pneumonia, there was a small proportion with positive bacterial microbiology results. Patients treated with antibiotics had more severe pneumonia and more often needed supplementary oxygen. Those treated with antibiotics also had more diarrhoea. Antibiotic treatment did not result in a lower subsequent ICU admission and were not independently associated with lower mortality. Although C-reactive protein levels showed acceptable discrimination based on AUC results, the sensitivity and specificity of cut-off points identified by the Youden index were poor. 

A meta-analysis reported that the respiratory bacterial co-infection rate at presentation of COVID-19 pneumonia was 3.5%, with a superinfection rate of 14.3%. Among studies that reported antibiotic prescribing, 71% of patients received antibiotics [10]. The trend of more antibiotic courses started compared to infections was similarly reported in a recent cohort of COVID-19 pneumonia (31, 19.1%) infections, with 24 bacterial, 3 fungal and 5 viral infections diagnosed but with antibiotic and antifungal treatments being administered in 71 out of 162 patients (43.8%) [11]. The majority of these studies reported only microbiologically confirmed co-infections. Although our cohort also included clinically diagnosed bacterial infections, which were not always microbiologically confirmed, there was still a higher proportion of patients receiving antibiotics compared to patients clinically assessed with co-infections. A recent guideline recommended that antibiotics should be stopped when representative sputum and blood cultures and urinary antigen tests taken before empiric antibiotics show no bacterial pathogens [7].

Patients who were ill were more likely to receive antibiotic treatment. There was higher mortality among those who received antibiotics in our cohort. However, antibiotic treatment did not result in a lower subsequent ICU admission rate and was not independently associated with reduced mortality. It is understandable that healthcare providers would err on the side of antibiotic treatment, especially in the hospitalised and the critically ill. However, it is important to be reminded that antibiotics are not without side effects. Patients treated with antibiotics were more likely to have diarrhoea. There are concerns that increased antibiotic use during the COVID-19 pandemic could lead to worsening of the antimicrobial resistance pandemic [12]. Evaluating the use of antibiotics in COVID-19 pneumonia patients will be an important step to mitigate drug toxicities and antimicrobial resistance. International guidelines recommend that cultures be obtained prior to antibiotics and that therapy be accessed daily for de-escalation [5,6,7]. When microbiology cultures are negative, antibiotics should be discontinued [12]. The majority of our patients were treated with an “access” group of antibiotics (e.g., amoxicillin–clavulanate) as recommended by WHO guidelines [13].

Compared to white blood cell count and procalcitonin level, the C-reactive protein level was the best performing inflammatory marker for predicting bacterial infection, even though its sensitivity and specificity were low. In one of the first reports of COVID-19 pneumonia, patients with severe disease had a median C-reactive level protein of 47.6 (IQR 20.6–87.1) mg/L compared to 34.2 (IQR 12.5–67.4) mg/L [14]. The median C-reactive protein level of those admitted to the ICU was 169 (IQR 111–324) mg/L [15]. The C-reactive protein level could potentially be used as another marker to help guide decisions for antibiotic treatment. However, in a cohort of ICU patients, the positive predictive value of procalcitonin > 1.00 ug/L for microbiologically proven secondary bacterial infections was 93%, while for C-reactive protein >50 mg/L and 150 mg/L the rates were 61% and 84%, respectively [16]. More studies on procalcitonin and C-reactive protein levels are needed to assess their utility in antibiotic decision-making in COVID-19 pneumonia.

Our study has several limitations. This was a cohort study from early in the pandemic, which may limit the generalisability of the results to current pandemic conditions. Not all patients underwent similar sets of microbiology investigations and we reported only the positive results. We were unable to study procalcitonin adequately, as this was not tested in all our patients. There was no independent assessment of bacterial co-infections or superinfections and the clinical diagnoses were collected from clinical charts by a team of pharmacists trained in antimicrobial stewardship as documented by treating physicians.

## 5. Conclusions

Overall, there was a higher proportion of patients treated with antibiotics compared to those documented with suspected or confirmed bacterial infections. Antibiotics were commonly started in patients who are more severely ill in COVID-19. However, antibiotic treatment did not prevent disease deterioration and was not associated with lower mortality. Patients given antibiotics were more likely to have diarrhoea. Inflammatory markers have limited clinical utility in identifying those who would benefit from antibiotic therapy. Clinical specimens for microbiological studies should be obtained prior to administration of antibiotics and therapy should be assessed daily for de-escalation or discontinuation.

## Figures and Tables

**Table 1:** Predictors for documented bacterial co-infection in patients diagnosed with COVID-19.

All COVID-19 Patients (*n* = 717)	Cut-Off	Sensitivity (%)	Specificity (%)	AUC (95% CI)
White blood cell count (10^9^/L)	≥7.2	64.8	78.4	0.767 (0.700–0.838)
Procalcitonin (ng/mL) ^a^	≥0.11	47.2	82.6	0.673 (0.570–0.777)
C-reactive protein (mg/L)	≥59.8	66.7	86.0	0.822 (0.756–0.887)
COVID-19 pneumonia (*n* = 278)				
White blood cell count (10^9^/L)	≥7.2	68.0	67.3	0.719 (0.639–0.798)
Procalcitonin (ng/mL) ^b^	≥0.11	48.6	73.5	0.607 (0.493–0.721)
C-reactive protein (mg/L)	≥73.2	68.0	69.7	0.720 (0.633–0.808)

^a^ Procalcitonin levels only available for 197 patients. ^b^ Procalcitonin levels only available for 133 patients. Cut-offs were determined by calculating the Youden index. AUC: area under the curve.

**Table 2:** Effects of antibiotics in the treatment of COVID-19 pneumonia on 30-day mortality.

	30-Day Mortality		
Characteristics	Univariate analysis Odd ratio (95% CI)	Multivariate analysis Odd ratio (95% CI)	*p*
Age, years, median, IQR	1.175 (1.100–1.259)	1.265 (1.070–1.495)	0.006
Male (%)	1.245 (0.356–4.356)	-	
Charlson’s score	1.536 (1.114–2.118)	0.860 (0.392–1.887)	0.707
Peak white cell count, 10^9^/L	1.016 (0.996–1.036)	-	
Peak C-reactive protein, mg/L	1.015 (1.008–1.021)	1.021 (1.002–1.039)	0.023
ICU admission at diagnosis of COVID-19 pneumonia (%)	5.298 (1.453–19.307)	0.024 (0.000–9.285)	0.219
Fraction of inspired oxygen, %, median, IQR	1.041 (1.019–1.064)	0.939 (0.863–1.021)	0.140
Mechanical ventilation	8.727 (2.0314–37.493)	470.581 (0.682–324642.500)	0.065
CURB-65 ^a^	3.897 (2.141–7.094)	1.351 (0.382–4.786)	0.470
Remdesivir	4.631 (1.139–18.836)	1.703 (0.135–21.467)	0.680
Documented suspected or confirmed bacterial infection	14.286 (3.640–56.061)	0.652 (0.053–7.967)	0.738
Antibiotic therapy	31.076 (3.904–247.400)	14.492 (0.533–393.875)	0.113

^a^ CURB-65 score for pneumonia severity. Only variables with *p* < 0.05 on univariate analysis were included in the multivariate mode. CI: confidence interval.

**Table 3:** Effects of antibiotics in the treatment of COVID-19 pneumonia on hospital mortality rates.

	Hospital Mortality		
Characteristics	Univariate analysis	Multivariate analysis	*p*
Age, years, median, IQR	1.163 (1.094–1.236)	1.291 (1.103–1.510)	0.001
Male (%)	1.622 (0.487–5.400)	-	-
Charlson’s score	1.515 (1.111–2.065)	0.748 (0.358–1.566)	0.442
Peak white cell count, 10^9^/L	1.016 (0.996–1.036)	-	-
Peak C-reactive protein, mg/L	1.015 (1.009–1.021)	1.026 (1.011–1.043)	0.001
ICU admission at diagnosis of COVID-19 pneumonia (%)	4.086 (1.176–14.194)	0.015 (0.000–6.929)	0.179
Fraction of inspired oxygen, %, median, IQR	1.036 (1.015–1.058)	0.930 (0.858–1.007)	0.075
Mechanical ventilation	6.927 (1.667–28.786)	618.086 (0.847–451009.000)	0.056
CURB-65 ^a^	3.381 (1.980–5.772)	1.100 (0.361–3.351)	0.867
Remdesivir	3.675 (0.935–14.439)	-	-
Documented suspected or confirmed bacterial infection	8.495 (2.650–27.234)	0.826 (0.074–9.221)	0.876
Antibiotic therapy	10.256 (2.739–38.402)	3.690 (0.240–56.811)	0.349

^a^ CURB-65 score for pneumonia severity. Only variables with *p* < 0.05 on univariate analysis were included in the multivariate mode. CI: confidence interval.

## Data Availability

The data presented in this study are available on request from the corresponding author.

## Supplementary Materials

The following are available online at https://www.mdpi.com/article/10.3390/antibiotics11020184/s1: Figure S1: Receiver operating characteristic (ROC) curves of white blood cell counts for the diagnosis of bacterial infection in patients with COVID-19 (a) and those with COVID-19 pneumonia only (b). Figure S2: Receiver operating characteristic (ROC) curves of procalcitonin levels for the diagnosis of bacterial infection in patients with COVID-19 (a) and those with COVID-19 pneumonia only (b). Figure S3. Receiver operating characteristic (ROC) curves of C-reactive protein levels for the diagnosis of bacterial infection in patients with COVID-19 (a) and those with COVID-19 pneumonia only (b). Table S1: Characteristics, treatment and clinical and adverse outcomes of COVID-19 pneumonia inpatients treated with and without antibiotics. Table S2: STROBE Statement—Checklist of items that should be included in reports of cohort studies.

## Abbreviations

COVID-19	Coronavirus disease 2019
RT-PCR	Reverse-transcriptase polymerase chain reaction
WHO	World Health Organization
ISARIC	International Severe Acute Respiratory and Emerging Infection Consortium
AUC	Area under the curve
ICU	Intensive care unit
